# Editorial: Rehabilitation and alternative medicine in the healthcare for chronic rheumatic pain disorders

**DOI:** 10.3389/fmed.2025.1586105

**Published:** 2025-03-21

**Authors:** Tieh-Cheng Fu, Nancy E. Lane, Si-Huei Lee, Juei-Chao Chen, Sheng-Feng Hsu, Ching-Mao Chang

**Affiliations:** ^1^Department of Physical Medicine and Rehabilitation, Chang Gung Memorial Hospital, Keelung, Taiwan; ^2^Division of Cardiology, Department of Internal Medicine, Heart Failure Research Center, Chang Gung Memorial Hospital, Keelung, Taiwan; ^3^School of Medicine, College of Medicine, Chang Gung University, Taoyuan, Taiwan; ^4^Department of Medicine, U.C. Davis Health, Sacramento, CA, United States; ^5^Department of Physical Medicine and Rehabilitation, Taipei Veterans General Hospital, Taipei, Taiwan; ^6^School of Medicine, National Yang Ming Chiao Tung University, Taipei, Taiwan; ^7^Department of Statistics and Information Science, Fu Jen Catholic University, New Taipei, Taiwan; ^8^Graduate Institute of Acupuncture Science, China Medical University, Taichung, Taiwan; ^9^Department of Chinese Medicine, China Medical University Hospital, Taipei, Taiwan; ^10^Center for Traditional Medicine, Taipei Veterans General Hospital, Taipei, Taiwan; ^11^School of Chinese Medicine, College of Medicine, National Yang Ming Chiao Tung University, Taipei, Taiwan; ^12^Institute of Traditional Medicine, College of Medicine, National Yang Ming Chiao Tung University, Taipei, Taiwan

**Keywords:** acupuncture, electroacupuncture, chiropractic, Tai Chi, yoga, rehabilitation, physical therapy, exercise

## Introduction

Chronic rheumatic pain disorders, including rheumatoid arthritis (1), osteoarthritis (2), fibromyalgia (3), and gout (4), represent a substantial burden on patients and healthcare systems due to their persistent nature, high prevalence, and impact on functional mobility (5). While conventional pharmacotherapy, such as disease-modifying antirheumatic drugs (DMARDs), corticosteroids, and biologic agents, remains a cornerstone of treatment, limitations in efficacy, long-term safety concerns, and potential adverse effects necessitate the exploration of adjunctive therapies (6). Consequently, there has been an increasing emphasis on rehabilitation and alternative medicine modalities (7), including evidence-based physical therapy interventions (8, 9), acupuncture (10), mind-body approaches (11) such as mindfulness-based stress reduction, and integrative medicine (12–15) strategies that combine conventional and complementary methodologies.

To provide a structured perspective on the various studies included in this Research Topic, we have categorized them into three major domains: Physical Therapy and Manual Techniques, Traditional and Complementary Medicine, and Epidemiological and Research Trends. Each of these categories encapsulates a distinct approach to managing chronic rheumatic pain disorders, offering insights into evidence-based rehabilitation, integrative therapies, and research trends that shape future clinical applications.

## Epidemiological and research trends

Research trends in physical therapy for knee osteoarthritis (KOA) from 2013 to 2022 identified key thematic clusters and influential contributors, with Harvard University leading contributions in the field (Huang et al.). Aerobic exercise and lower limb strengthening were found to be effective, emphasizing the need for personalized exercise dosing and digital interventions to promote self-management. Future studies should incorporate rigorous trials to validate these approaches. A cross-sectional study in Poland examined occupational risk factors for nonspecific neck pain among healthcare workers, identifying prolonged static postures, ergonomic deficiencies, and psychological stress as key contributors (Citko et al.). These findings highlight the need for ergonomic and rehabilitative interventions to mitigate occupational risks. Another bibliometric analysis assessed global trends in dry needling from 2004 to 2024, highlighting increasing academic interest but a lack of large-scale, high-quality trials validating its efficacy (Wang et al.).

These studies provide a comprehensive assessment of the evolving landscape of rehabilitation and alternative medicine research. By examining global research trends, occupational risk factors, and emerging therapeutic modalities, they highlight key areas for clinical advancement and underscore the necessity for evidence-based validation. The findings emphasize the importance of interdisciplinary collaboration and methodological rigor in ensuring the effective integration of physical therapy, ergonomic interventions, and alternative treatments into mainstream healthcare practices. Moving forward, continued efforts in high-quality clinical trials, international research cooperation, and digital rehabilitation innovations will be essential for optimizing patient outcomes and expanding the accessibility of evidence-based rehabilitative care.

## Physical therapy and manual techniques

A review on KOA management emphasized pharmacologic and nonpharmacologic approaches, including platelet-rich plasma (PRP), stem cell therapy, exercise, and multimodal pain management, advocating for integrative strategies to optimize patient outcomes (Shtroblia et al.). An umbrella review evaluated PRP efficacy for KOA using nine meta-analyses encompassing 46 datasets, with mixed findings regarding its effectiveness in pain relief and mobility improvement (Yu et al.). Variability in PRP preparation and patient selection contributed to these inconsistencies, emphasizing the need for standardized methodologies and long-term outcome assessments. Sartori et al. conducted a randomized controlled pilot trial to evaluate ultrasound-guided percutaneous peripheral nerve stimulation (pPNS) targeting the gluteal nerves in individuals with chronic knee pain. The intervention improved hip extension power, suggesting potential benefits for knee function and pain management. Further research is needed to confirm these findings (Sartori et al.).

While promising, larger studies are needed to confirm its clinical applicability. A retrospective study assessed Pridinol Mesylate combined with rehabilitation in elderly patients with spondyloarthritis-related low back pain, showing superior pain reduction and functional improvement compared to monotherapy with either intervention alone (Lauricella et al.). Further trials are necessary to confirm long-term efficacy. A randomized trial compared the efficacy of Extracorporeal ShockWave Therapy (ESWT) and mesotherapy for myofascial pain syndrome, concluded that ESWT provided greater pain relief and improved mobility (Scaturro et al.). Another study evaluated high-frequency laser therapy (HFLT) for cervical disk herniation, demonstrating comparable efficacy to physiotherapy, suggesting it as a noninvasive alternative (Kuculmez et al.).

These studies underscore the growing significance of evidence-based rehabilitation techniques in managing chronic pain conditions. By incorporating regenerative medicine, neuromodulatory interventions, high-frequency laser therapy, and ESWT, these approaches contribute to a comprehensive, multimodal treatment framework aimed at enhancing pain relief, restoring function, and improving overall patient wellbeing. The findings further highlight the necessity of rigorous clinical validation and interdisciplinary collaboration to refine and optimize rehabilitation methodologies for musculoskeletal and rheumatic disorders, ensuring their effective integration into mainstream clinical practice.

## Traditional and complementary medicine

A randomized trial investigated the effects of Yijinjing, a traditional Chinese exercise, on rheumatoid arthritis (RA)-related hand dysfunction, reporting significant improvements in hand mobility, grip strength, and quality of life (Chang et al.). Although promising, further research is required to establish long-term efficacy and determine optimal exercise regimens. A case report explored the application of traditional Chinese manual therapy in a 10-year-old patient with adolescent idiopathic scoliosis, showing improvements in spinal alignment and pain reduction over 18 months (Zhu et al.). Larger studies are needed to confirm its efficacy as a conservative treatment option. A case series examined acupuncture for linezolid-induced peripheral neuropathy in multidrug-resistant tuberculosis, documenting pain relief and functional recovery (Mo et al.). However, larger controlled trials are required to validate these findings. A case report integrated graded exercise with motion-style acupuncture therapy for a patient with failed back surgery syndrome and major depressive disorder. This intervention demonstrated physical and psychological improvements (Kim et al.) suggesting future studies should explore if this intervention has broader applicability. A meta-analysis reviewed osteopathic craniosacral therapy (CST) for chronic pain and mobility disorders, and it demonstrated some benefits in chronic somatic pain but raising concerns about methodological rigor and placebo effects (Amendolara et al.).

These studies highlight the integration of traditional therapeutic modalities with modern rehabilitative techniques, demonstrating their potential for neuromuscular reconditioning, pain modulation, and functional improvement. By incorporating acupuncture, structured therapeutic exercise, craniosacral therapy, and manual interventions, these complementary approaches provide holistic, patient-centered treatment options for managing chronic pain and musculoskeletal disorders. While preliminary findings suggest promising benefits, further rigorous, high-quality research is essential to establish standardized protocols, validate long-term efficacy, and optimize their integration into contemporary clinical practice.

## Future perspectives

As rehabilitation and alternative medicine therapies evolve, future research should focus on optimizing evidence-based integrative treatment strategies for chronic rheumatic pain disorders. Key areas for advancement include standardizing physical therapy protocols, enhancing neuromodulatory techniques, and refining traditional therapeutic modalities such as acupuncture and osteopathic manipulation. Personalized treatment paradigms based on patient-specific factors (e.g., inflammatory biomarkers, biomechanical deficits, and central pain sensitization) could enhance therapeutic precision and long-term recovery. Differentiating pain phenotypes, such as inflammatory vs. mechanical pain, may also improve individualized interventions. Digital health solutions offer new opportunities to refine rehabilitation. AI-driven models analyzing biomechanical data from wearable devices can optimize rehabilitation exercises, while tele-rehabilitation platforms enable remote monitoring. Integrating machine learning algorithms may improve patient-specific treatment responses, fostering a precision medicine approach to pain management. International collaborations will be essential in bridging traditional healing practices with modern medicine to enhance patient outcomes.

Despite promising findings, several challenges remain. Heterogeneity in protocols, patient selection, and outcome measures complicates standardization. Placebo effects in complementary medicine require sham-controlled trials to validate efficacy. Future research should adopt harmonized methodologies to ensure reproducibility and external validity. While regenerative and complementary therapies gain recognition, cost-effectiveness and accessibility remain concerns. Many treatments are expensive and lack reimbursement, limiting patient access. Health policy initiatives should expand insurance coverage, conduct cost-effectiveness analyses, and integrate these therapies into multidisciplinary pain management programs to maximize their public health impact.

Successful integration of physical therapy, neuromodulatory techniques, and complementary medicine requires structured clinical pathways. Establishing standardized treatment algorithms based on patient phenotyping (e.g., pain severity, functional impairment, prior treatment response) could enhance clinical precision. Multidisciplinary collaboration among rheumatologists, physiotherapists, and integrative medicine practitioners will be key to advancing effective and sustainable chronic pain management.

## Conclusion

This Research Topic highlights the diverse and evolving role of rehabilitation and alternative medicine in the management of chronic rheumatic pain disorders. The included studies demonstrate the therapeutic potential of physical therapy, manual interventions, acupuncture, neuromodulatory approaches, and osteopathic techniques in improving pain relief, functional mobility, and quality of life. While these findings emphasize the value of multimodal, patient-centered treatment strategies, further high-quality clinical trials and interdisciplinary collaborations are necessary to establish standardized, evidence-based protocols for broader clinical application. Moving forward, an integrative, personalized approach that combines conventional and complementary therapies will be essential in addressing the complex challenges of chronic pain management, ultimately leading to more effective, holistic, and sustainable healthcare solutions.
